# High IgE sensitization to maize and rice pollen in the highlands of Madagascar

**DOI:** 10.11604/pamj.2014.19.284.4654

**Published:** 2014-11-15

**Authors:** Ramavovololona, Hélène Sénéchal, Ange Andrianarisoa, Vololona Rakotoarimanana, Dominique Godfrin, Gabriel Peltre, Pascal Poncet, Jean-Pierre Sutra

**Affiliations:** 1 Sciences University, Biology and Plant Ecology Department, BP 906, Antananarivo 101, Madagascar; 2 Armand Trousseau Children’s Hospital, Biochemistry Department, Allergy & Environment, 26 avenue du Dr Arnold Netter, 75571 Paris cedex 12, France; 3 Inserm, 101 rue de Tolbiac, 75013 Paris, France; 4 Medical University, Medecine and Medical Specialities Department, BP 375, Antananarivo 101, Madagascar; 5 Laboratory of Scientific Police, 31 Avenue Franklin Roosevelt, 69130, Ecully, France; 6 CNRS, 75794, Paris cedex 16, France; 7 Pasteur Institute, Infections & Epidemiology Department, 25-28 rue du Dr Roux, 75724, Paris cedex 15, France

**Keywords:** IgE binding proteins, Madagascar, maize, rice, pollen allergy, urban and peri-urban agriculture

## Abstract

**Introduction:**

Maize and rice are two crops constituting the main food supply in many under-developed and developing countries. Despite the large area devoted to the culture, the sensitization to the pollen from these plants is reported to be low and often considered as an occupational allergy.

**Methods:**

Sixty five Malagasy pollen allergic patients were clinically and immunochemically investigated with regard to maize and rice pollen allergens. Pollen extracts were electrophoretically separated in 1 and 2 dimensions and IgE and IgG reactivities detected upon immunoblotting.

**Results:**

When exploring the sensitization profile of Malagasy allergic patients to maize and rice pollen, it appears that a high proportion of these patients consulting during grass pollinating season were sensitized to both pollen as revealed by skin prick testing (62 vs. 59%) and IgE immunoblotting (85 vs. 40%). Several clinically relevant allergens were recognized by patients’ serum IgE in maize and rice pollen extracts.

**Conclusion:**

The high levels of maize and rice pollen sensitization should be related, in this tropical region, to a specific environmental exposure including i) a proximity of the population to the allergenic sources and ii) a putative exacerbating effect of a highly polluted urban atmosphere on pollen allergenicity. Cross-reactivities between wild and cultivated grasses and also between rice and maize pollen are involved as well as some specific maize sensitizations. The presence of dense urban and peri-urban agriculture, in various African regions and worldwide, could be a high environmental risk factor for people sensitive to maize pollen.

## Introduction

The highlands constitute, in Madagascar, the main populated area with the presence of the capital town - Antananarivo (around 1.85M inhabitants) - and of Antsirabe (currently around 200, 000 inhabitants). Beside traditional rural agricultural systems, the food supply for such large cities includes, now for decades, peri-urban and urban agricultural zones, as already described in other towns from developing countries [1–3]. Rice (Oryzeae) is the main crop in the highlands as well as in other regions of Madagascar. It arrived on this land with the Austronesians who settled as primal inhabitants in the first millennium of the Christian era [4], whereas maize, of American origin, was introduced quite later through Europe-Africa trade exchanges. The presence of maize culture was attested in the island in the first part of the 17^th^ century [5]. Regarding rice cultivation, the irrigated one is the most important, covering more than 80% of all areas under this cereal. The maize (Panicoideae, Maydeae tribe), used for local food supply is the second cereal crop in Madagascar. Among the different varieties, two of them - Meva and Volasoa - are cultivated by around 40% of the farmers (www.inter-reseaux.org). The maize is mostly cultivated at proximity of the population and the rice is grown in irrigated paddy fields, most often away from inhabitants in the highlands of Madagascar.

Despite its increasing worldwide trends, allergy remains poorly studied in Madagascar [6, 7]. In the same West Indian Ocean and South African zones, some studies have reported that the urban population is now often affected and regularly treated [8–10]. Among respiratory allergic diseases, allergy to grass pollen represents an important part because grasses are widely distributed. Aerobiological studies in Antananarivo showed that grass pollen is determinant in the urban atmosphere [11]. Sensitization to rice and maize pollen has been clinically investigated [12, 13]. They are often considered as an occupational allergy in farmers and field workers [14–16] or the result of the documented cross-reactivities with wild grasses [17–19]. Reported allergens for pollen of these 2 crops are listed in Table 1. Ten allergens have been described in maize and 7 of them, belonging to the same protein families, were also found in rice pollen. The major allergens β-expansins (groups 1-3 of grass pollen allergens) [20–22], profilin (group 12) [23] and polygalacturonase (group 13) [24] were characterized both in maize and rice pollen.

**Table 1:** Previously reported allergens from maize and rice pollen. (http://www.allergome.org, http://www.allergen.org, http://www.allerdata.com/ and http://www.meduniwien.ac.at/allergens/allfam/)

Group “Nbr”	Allergen name		Protein family	Mr (kDa)	pI
	*Zea mays*	*Oryza sativa*			
1	Zea m 1	Ory s 1	• expansin	28-32	5.1-9.0
2	Zea m 2	Ory s 2	• expansin	10-12	5.0-9.6
3	Zea m 3	Ory s 3	• expansin	10-12	9.0-9.5
4	Zea m 4	*(not described)*	Reticulin oxidase	57-58	8.7
5	Zea m 5	*(not described)*	Ribonuclease	28-35	*(unknown)*
7	Zea m 7	Ory s 7	Calcium Binding Protein	6	8.6
11	Zea m 11 (Zm13)	Ory s 11	Trypsin Inhibitor, Ole e 1-like	9-10	5.0
12	Zea m 12*	Ory s 12*	Profilin	14	4.4-5.0
13	Zea m 13	Ory s 13	Polygalacturonase	55	6.8-7.6
22	Zea m 22	*(not described)*	Enolase 2	48	5.7
23	Zea m 23	Ory s 23	Cyn d 23-like	26	8.7

* Allergen in IUIS

In order to explore the extent of respiratory diseases related to allergy to grass pollen in Madagascar, an epidemiological study was carried out on patients consulting for respiratory disorders in two different centers of Antananarivo [11]. On the basis of a standardized questionnaire, clinical symptoms, skin prick test and correlations of symptoms with pollen airborne content and/or documented pollinating season, 65 allergic patients were selected to quantitatively and qualitatively evaluate some clinical and immunochemical parameters associated to grass pollinosis. In a previous report, we published the results related to 6 selected wild grass pollen and showed that, besides cross-reactivities, sensitization is mainly directed against the local tropical dominant wild grass, *Rhynchelytrum repens* [7]. To give a complete picture of the prevalence of allergy to grass pollen in Madagascar our results are now extended to cultivated grasses, maize and rice, that show overlapping pollinating periods with wild grasses. The level of sensitization to the pollen of these two main crops was clinically and immunochemically evaluated and was found to be high. The proximity of cultivation areas, close to inhabitants, and/or a highly polluted atmosphere, could be the crucial environmental risk factors in the development of such allergic symptoms in this region.

## Methods

This study was conducted in accordance with the principles of the declaration of Helsinki, with institutional approval (from both University of Antananarivo, Madagascar and Hôpital Civil, Strasbourg, France). Written informed consent has been obtained from all patients.

### Patient recruitment and patient sera

A previous comprehensive epidemiological study was carried out in the Lung and Allergy Department of the “Institut d'Hygiène Social” and the “Infirmerie de la Gendarmerie du Toby Ratsimandrava” in Antananarivo (Madagascar) on about 1500 individuals suffering from respiratory disorders (consultations and data files analysis). The follow up during 2 years included the record of clinical symptoms such as respiratory infections, asthma, rhinitis, conjunctivitis, rhino-conjunctivitis, dyspnea but also dermatological disorders such as dermatitis, eczema and urticaria [11]. From this initial cohort, a sub cohort of 65 patients was selected on the basis of (i) the correlation of clinical symptoms with the grass pollinating season. (ii) skin prick test (reported in [7] and in this paper).and (iii) a standardized questionnaire prepared by physicians with extensive experience in allergic diseases. All grass pollen allergic patients (PAP), 28 males and 37 females, mean age 33 year-old (range: 3 to 65), participated voluntarily in the study. The main symptoms for these patients were rhinitis, conjunctivitis and/or asthma during the flowering season. Some PAP showed food allergy symptoms such as diarrhea, nausea, vomiting associated to rashes. The sera used in this survey are the same than for our previous study [7]. Two additional sera from grass and birch sensitized Caucasian/European patients born in Strasbourg area, (France) were also used [7, 25]. These patients had never been in Madagascar. A serum from a non-allergic non-atopic individual was used as control.

### Maize and rice pollen material

The pollen was collected directly from the flowers of the 2 crops between March and April. The *Zea mays* pollen used for most of the experiments of our study was from a *Meva* cultivar. This variety grows all around the city of Antananarivo and is one of the most cultivated in the island. It is a polyhybride registered as N° 374. This synthetic construction combining 5 South African and a local yellow lines variety has been selected for its resistance to leaves disease and for its adaptation to acidic soils. Its culture is agronomically adapted to an altitude ranging from 800 to 1500 m and to the climate of highlands. Five other varieties of maize pollen were used for 1D-isoelectrofocalisation (IEF) and immunoblot experiments. Four are from Madagascar: “*Paysanne*”, a local farmer variety, “*Volasoa*” -also called “*Los banös 8227*”- as well as 2 varieties, created by private companies, “*NTS*” and “*Tombotsoa*”. The last one is from Europe (Allergon AB, Sweden, W 2552). The rice pollen was a blend from several varieties among the three main seasonal types cultivated in the highlands locally called - from the 1^st^ to 3^rd^ seasonal one - respectively: vary aloha, vary vakiambaty and vary siha, including *indica* and *japonica* subspecies.

### Pollen extracts for skin prick tests (SPTs) and SPTs

Pollen extracts were prepared in the same way than in Ramavovololona et al [7]. Briefly: 1 mL of 0.9% NaCl, 0.3% NaH(CO_3_)_2_ was added to 100 mg of each pollen and obtained as previously described [25, 26]. SPTs were performed using these pollen extracts and tested on the 65 PAP for the 2 taxa. For the 2 Caucasian/European patients, the grass pollen extract was provided by Stallergènes Laboratories, (Antony, France). A negative control test with saline solution and a positive control test with histamine (1 mg/mL) were included in each set of SPT. SPT response was positively scored if the largest wheal diameter was greater than that was produced by the negative control and at least 70% greater than that was produced by the positive control.

### Pollen extracts for IEF and immunodetections

Water soluble extracts were obtained by incubating 30 mg of fresh pollen for 1h at 22°C in 150µL sterile water followed by a centrifugation at 4°C for 15 min at 10,000 g. The supernatants were kept at -20°C.

### 1D-IEF separation and 1D-immunodetection

IEF was performed in a 0.8% agarose gel (Isogel, FMC Rockland, Maine, USA) [27] containing 12% (w/v) sorbitol and 2% (v/v) of a mixture of carrier ampholytes (Servalyt^®^ pH 3-10, Serva, Heidelberg, Germany) on a flat bed electrophoretic chamber (Multiphor II, Amersham Biosciences, Uppsala, Sweden) cooled at 15°C according to the manufacturer's instructions. Standard proteins with a range of isoelectric point (pI) from 4.7 to 10.6 (BDH Electron, VWR International, England) were used as pI markers. After IEF, a part of the gel was stained with Coomassie blue. The other part of the gel was blotted on either nitrocellulose (NC) (Optitran BA-S 83, Schleicher & Schuell, Dassel, Germany) [7] or polyvinylidene fluoride membrane (PVDF, Immobilon TM, Millipore, Bedford, MA, USA) sheets. The membranes were then dried and cut into strips (3 mm width) that were incubated overnight at 22 °C with individual sera diluted at 1:10 [7]. Thereafter, the strips were incubated either with alkaline phosphatase (AP)-conjugated goat anti-human IgG (Sigma-Aldrich) diluted at 1:3000 or peroxidase (PO)-conjugated goat anti-human IgE (Sigma-Aldrich) diluted at 1:1000 in tris buffer saline with 1% defatted milk and 0.2% Tween 20 (TBS-M-Tw). After 3 washes with TBS-Tw and 3 washes with TBS, the IgG and IgE bindings were detected by chemiluminescence (Amersham ECL kits, GE Healthcare, Uppsala). For PVDF membranes used in IEF immunoblotting of the different maize varieties pollen extracts, the first steps were identical to what was described above. But after incubation with serum and washing steps, membranes were incubated with AP-conjugated goat anti-human IgE (Sigma-Aldrich) diluted at 1:700 in TBS-M-Tw (2h at 22°C). The AP activity was detected using 5-bromo-4-chloro-3-indolyl phosphate and nitroblue tetrazolium (Sigma) in 0.1 M tris buffer pH 9.5.

### Two-dimensional gel electrophoresis (2D)-immunodetection

For 2D separation, Zea mays pollen extract was submitted to an IEF separation performed in a polyacrylamide gel 5%C (GE Healthcare) containing 5% v/v Ampholine pH 3.5-9.5 as described above. After the IEF separation, 4 mm-wide strips of the focused gel were cut, incubated in the equilibration buffer (120mM Tris pH 6.8, 12% SDS) and submitted to SDS-PAGE separation on an 8-18% gradient gel (GE Healthcare). Standard proteins with a range of relative molecular mass (Mr) from 14.4 to 94 kDa (GE Healthcare) was used as Mr markers. One of the 2D gels was silver-stained according to Blum et al. [28] in order to visualize all proteins and the other ones were electroblotted onto a bromide cyanogen-activated nitrocellulose membrane (NCa) [29] using a semi-dry Novablot apparatus (Amersham Biosciences) according to the manufacturer's instructions for 1h at 1 mA/cm^2^. The NCas were then dried, saturated with phosphate buffer saline - 0.3% tween 20 and treated as described above. Then, membranes were incubated with AP-antibodies against either human IgG or IgE.

### Statistical analysis

The statistical significance of differences in data was evaluated using Fisher's exact test.

## Results

**Figure 1:**
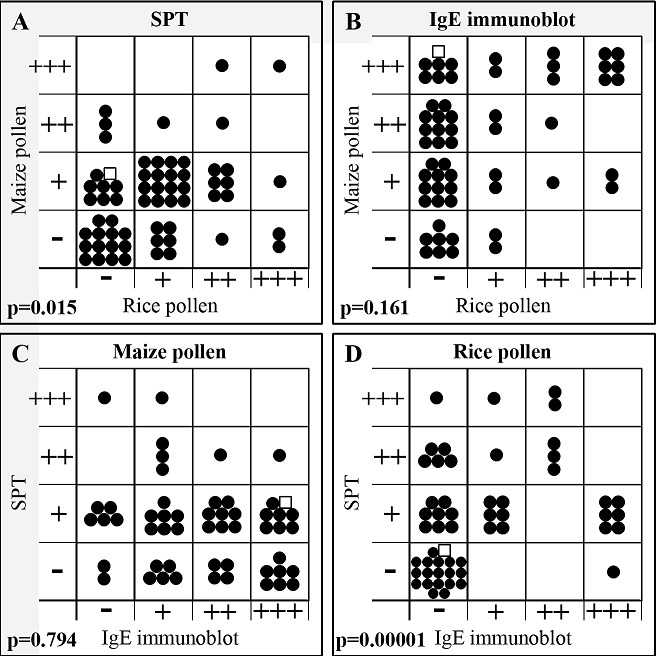
Correlations between IgE reactivities studied by SPT or IgE immunoblots for maize and rice pollen extracts. “Each dot (•) represents the result obtained with one patient; ✩: patient #60;”; A: SPT maize vs SPT rice; B: IgE immunoblot maize vs IgE immunoblot rice; C: SPT maize vs IgE immunoblot maize; D: SPT rice vs IgE immunoblot rice. SPT response was scored as + positive, if the largest wheal diameter was comprised between 70 to 80% greater than that was produced by the positive control, ++ between 80 to 90% and +++ between 90 to 100% Results of IgE immunoblot (see reactivity figure 2) are semi quantitatively expressed according to the intensity of the reaction as: -: negative; + positive low; ++ medium; +++ very high.

Clinical data of studied PAP are shown in Table 2, Table 3. Patients were mainly suffering from rhinoconjuntivitis (72%), 53% from asthma and 15% urticaria. Thirty one patients out of 65 (47.7%) exhibited food allergy symptoms. Percentages of patients tested SPT-positive for maize or rice pollen extracts were both around 60% (Table 4) as compared to 71% for wild grasses. By SPT (Figure 1, A), 44% of patients were found co-sensitized to maize and rice pollen extract and among them, the majority (59%) had a weak positivity. The correlation between SPT to maize and rice pollen was statistically significant (p = 0.015).

**Table 2:** Clinical data of studied Malagasy PAP (part 1). ND: No Data available. Patients # 66 and 67 are Caucasian/European patients

Patient	Gender	Age	Symptoms‡	Food allergy†	SPT*		
Nbr					Maize	Rice	Wild grass§
1	M	36	RA	-	+ +	-	+ +
2	F	42	R	fish, soybean, grasshopper	+ +	+ +	+ +
3	F	16	R	-	-	+	+
4	F	36	R	-	+ + +	+ +	+ + +
5	F	42	RA	-	+	-	+
6	M	ND	ND	-	-	-	-
7	F	10	R	-	-	+	+ + +
8	M	30	R	-	ND	ND	ND
9	M	38	RU	hen egg, seafood, rice	-	-	-
10	F	16	RA	edible oil, cooked pork meat, fish, papaya, pear, plum, apple	+	+ +	+ +
11	F	ND	ND	-	-	-	+ +
12	M	34	RA	cereal, sea food, honey cake	+	+	+ +
13	F	ND	RCA	-	-	+ + +	+ +
14	M	22	RCA	manioc, orange, tangerine	-	+ +	+
15	F	27	A	sea food	+	+	+
16	M	44	A	-	+	-	+
17	F	47	RCU	edible oil	+	+ +	+ +
18	M	40	RCU	insect larva	+ +	-	-
19	F	41	E	-	-	-	-
20	M	30	RA	ND	+	-	+ +
21	M	41	A	-	-	-	-
22	M	ND	U	-	+	-	-
23	F	20	RA	-	+ + +	+ + +	-
24	F	10	R	-	-	+	-
25	F	32	RA	sea food	+	+	+
26	F	ND	ND	ND	-	-	-
27	F	38	RCAU	sea food, eggplant	+	+	-
28	F	47	R	-	-	+	+
29	F	50	AU	-	+ +	-	+ +
30	M	64	AU	-	+	-	+
31	M	48	RA	manioc (flour and leaves)	+	+	+

* SPT to pollen extracts semi quantitatively expressed according to the measured size of the wheal diameter.

- Negative

+ 70 to 80% greater than that was produced by the positive control

++ 80 to 90%

+ + + 90 to 100%

† without food allergy

‡ R: Rhinitis; A: Asthma, C: Conjunctivitis; U: Urticaria; E: Eczema; D: Dyspnea

§ The highest positivity is indicated out of the 6 following tested wild grass pollen: *Rhynchelytrum repens, Panicum maximum, Pennisetum polystachion, Imperata cylindrica, Cynodon dactylon, Aristida rufescens*

**Table 3:** Clinical data of studied Malagasy PAP (part 2). ND: No Data available. Patients # 66 and 67 are Caucasian/European patients

Patient Nbr	Gender	Age	Symptoms‡	Food allergy†	SPT*		
					Maize	Rice	Wild grass§
32	M	13	U	sea food	+	+	+
33	F	23	A	sea food, papaya	+	+ +	+ +
34	M	44	U	-	-	-	-
35	M	49	RA	manioc (flour and leaves)	ND	ND	-
36	M	46	R	-	+	+	+ +
37	F	44	AU	rice	+	+	+
38	F	40	RCU	-	-	+ + +	+ + +
39	M	42	RA	-	-	-	-
40	M	65	RAU	sea food	-	-	-
41	M	25	R	peach	+	+	+ +
42	F	28	ND	sea food, fish	-	-	-
43	M	ND	R	sea food	+	+ +	+ + +
44	F	50	RA	sea food, manioc leaves	-	-	+
45	M	ND	RA	-	ND	ND	+
46	F	35	R	Beer, wine	+	+	-
47	F	35	R	-	+	-	-
48	F	33	RCU	sea food, avocado	+	+	-
49	M	44	A	-	ND	ND	ND
50	M	11	R	chocolate	+	+ +	+ + +
51	M	51	U	-	+	+	+
52	F	38	RA	manioc leaves, garlic, fish, cabbage, legumes	+	+ +	+ +
53	F	16	RCAU	sea food	+	+	+
54	F	10	RA	manioc leaves, fish	-	+	+
55	F	11	RA	onions, pepper	-	-	+
56	F	37	RA	chocolate	+ +	+	+ + +
57	F	44	R	sea food, stock fish, dried legumes	+	-	+
58	F	28	R	-	+	+	+
59	F	15	A	sea food, stock fish	+	+	+
60	M	3	RA	-	+	-	+
61	M	49	ND	-	+	-	+
62	M	9	D	-	-	+	+
63	M	39	R	-	-	-	+
64	F	10	A	sea food, mayonnaise	+	+	+
65	M	46	A	-	+	+ + +	+ + +
66	F	33	RC	apple, peach, hazelnut, celery, carrot	ND	ND	+ +
67	M	40	RC	apple, peach, walnut, potato	ND	ND	+

* SPT to pollen extracts semi quantitatively expressed according to the measured size of the wheal diameter.

- Negative

+ 70 to 80% greater than that was produced by the positive control

++ 80 to 90%

+ + + 90 to 100%

† -: without food allergy

‡ R: Rhinitis; A: Asthma, C: Conjunctivitis; U: Urticaria; E: Eczema; D: Dyspnea

§ The highest positivity is indicated out of the 6 following tested wild grass pollen: *Rhynchelytrum repens, Panicum maximum, Pennisetum polystachion, Imperata cylindrica, Cynodon dactylon, Aristida rufescens*

**Table 4:** Percentages of PAP tested positive for SPT and Ig immunoblots (IgG and IgE) for the 2 crops pollen extracts (*Zea mays* and *Oryza sativa*). Between brackets: number of tested patients

	Pollen	
	*Zea mays*	*Oryza sativa*
**SPT**	63.9% *(61)*	59.0% *(61)*
**IgE**	84.6% *(57)*	39.7% *(63)*
**IgG**	100% *(59)*	75.4% *(65)*

**Figure 2:**
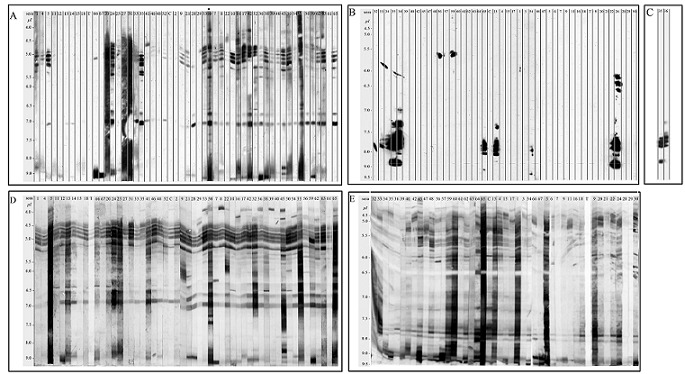
Most representative patterns from 1D IEF immunoblots for the 2 studied crop pollen extracts. Each strip corresponds to the IgE reactivity from individual allergic patient serum: (A) 49 sera were tested with blotted Zea mays pollen extract; (B) 42 sera were tested with blotted Oryza sativa pollen extracts and (C) Sera # 35 and #36 tested with *Oryza sativa* pollen extract; results of chimioluminescence obtained after a five-fold reduced exposure time. The IgG reactivity from allergic patient serum were tested (D) with blotted *Zea mays* pollen extract and (E) with *Oryza sativa* pollen extract; Strips #66 and #67: 2 European/Caucasian allergic patient sera; c: strip incubated with a non-allergy individual serum as control; T: negative control strip with buffer; at the top: serum number; on the left side: pI values

The specificity of seric IgE (Figure 2) were evaluated by immunoblot after separation of protein extract with IEF which has the advantage over other electrophoretic separation techniques to provide separations of proteins in their native conformation. Immunoblotting experiments showed that maize pollen proteins elicited IgE antibodies (Ab) (Figure 2, A) in 84.6% of patients and IgG Ab (Figure 2, D) in all of them. In contrast, IgE (Figure 2, B,C) and IgG (Figure 2, E) responses against rice pollen proteins were found only in 39.7% and 75.4% of patients, respectively (Table 4). Interestingly, in some patient sera, about 12%, the Ab response to rice pollen protein seemed restricted to IgE isotype since no specific IgG isotype could be detected (data not shown). Moreover, 19 patient sera (33%) showed IgE reactivity against proteins from both pollen extracts (Figure 1, B), but the majority (51%) of patient sera only to maize pollen extract. In contrast to what was found by SPT, the comparison of IgE reactivity against maize and rice pollen evaluated by IgE immunoblot did not reveal any correlation (p = 0.161). Furthermore, the comparison of SPT and immunoblot to evaluate the IgE reactivity showed no correlation for maize pollen extract (p = 0.794) and a very significant correlation for rice pollen extract probably because of a higher number of double negative sera (36%) (p = 0.00001) than for maize pollen (Figure 1, C, D).

The 1-D immunoblot results of each of the 2 crops pollen extracts (Figure 2) highlighted specific IgE-binding protein patterns. While no IgE reactivity was observed with a control serum (Figure 2, A) from a non-allergic individual, but having IgG reactivity (Figure 2, D), patients’ allergic sera showed IgE reactivities against several maize proteins in a wide pI range. Two IgE-binding protein acidic zones, pI 4.6-5.5, the most frequently recognized and 5.5-6.0 can be visualized. These two zones might correspond to the allergens Zea m 1 and 2 (β-expansins) and the maize profilin (Zea m 12) (pI 4.4-5.0) as described in Table 1. A 3^rd^ neutral zone (pI 6.6-7.1) showed also a strong IgE signal for several patient sera (Figure 2, A) and might correspond to isoforms of Zea m 13, the maize pollen polygalacturonase. IgG reactivities were also observed against several maize proteins in a wide pI range (Figure 2, D). By comparison, IEF immunoblots of the rice pollen extract exhibited less heterogeneity of IgE binding (Figure 2, B), but IgG reactivity against this extract was very heterogeneous (Figure 2, E). For the IgE reactivity, the pI range was more restricted and most of the sera gave positive signals in the neutral-basic zone, from pI 7 to 9.5 (Figure 2, B, C), consistent with already described allergens Ory s 1, 2, 3, 7 and 23 (Table 1). A few patient sera showed IgE binding to proteins with pI between 5.5 and 6.5 as well as in pI range from 6.0 to 6.8 (serum #24), that could correspond to isoforms of Ory s 1. IgE reactivities against basic allergens (pI > 9.0), were found only in the maize pollen extract and could correspond to Zea m 3. Sera #66 and #67, from European patients, (called A and B in our previous work [7]) showed a strong IgE binding to basic allergens in maize extract (Figure 2, A) whereas no IgE reactivity was found in rice extract (Figure 2, B).

**Figure 3:**
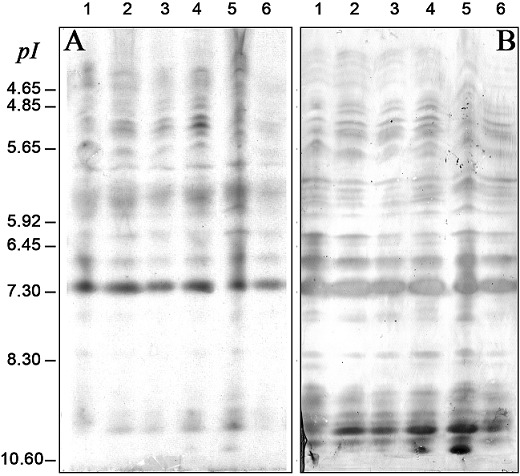
1D IEF Coomassie blue staining (A) and 1D IEF immunoblots (B) for the pollen of 6 varieties of Zea mays. (1): “Paysanne”, (2): “Volasoa”, (3): “NTS”, (4): “*Meva*”, (5): Europe, (6): “Tombotsoa”. The proteins from the pollen extracts separated by IEF and blotted were incubated with the serum #60 showing a high IgE reactivity (see Figure 1). pI values are indicated on the left side

**Figure 4:**
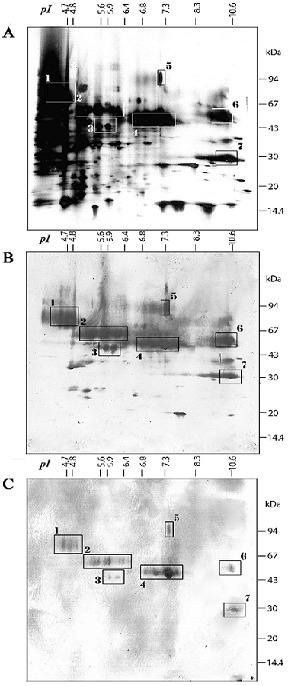
2D analysis of the water-soluble proteins from Zea mays pollen. Pollen extract from Zea mays was submitted to an initial IEF separation followed by SDS-PAGE separations. The gels was either silver stained (A) or transferred on NCas and incubated with from pollen-sensitized patient serum #60. IgG (B) or IgE (C) binding were revealed using heavy chain specific Ab coupled to alkaline phosphatase followed by substrate. Boxes on each figure indicate groups of allergens recognized by IgE PAP. pI values (at the top) and Mr (kDa, on the right side) are indicated for each gel

Because of the heterogeneous IgE recognition patterns observed in 1D IEF immunoblot results for *Meva* cultivar maize pollen allergens, IgE binding profiles against 4 other local varieties of maize and an European one (Figure 3) were compared. Patient's serum #60 was used because of its highly heterogeneous IgE binding pattern against the *Meva* variety pollen extract (Figure 2, A). Both total protein (Figure 3, A) and IgE binding (Figure 3, A) patterns of the 6 varieties were close except in the very basic zone where only *Meva* and European varieties showed a strong IgE reactivity. Consequently, with regard to allergenicity, the *Meva* variety (lanes 4) is the closest to the European cultivar (lanes 5). To characterize the protein and antigenic repertoire of *Meva* variety maize pollen extract, 2D separation was performed. The gels were either silver stained for total protein visualization (Figure 4, A) or electroblotted onto NCas for subsequent characterization of IgG (Figure 4, A) or IgE (Figure 4, C) binding proteins with patient's serum #60 (already studied in 1D immunoblot experiments, see Figure 2, A strip indicated by a dot). About 200 protein spots were detected upon silver staining, with a broad distribution in pI (from 4.0 to 11) and in Mr (from 14 to 100 kDa) giving patterns in spot series suggestive of numerous isoforms (Figure 4, A). Antigens recognized by IgG of patient #60 (Figure 4, B) are much more diversified than the sole allergens (IgE binding proteins, Figure 4, C). They are mainly located in Mr > 30 kDa and covered the whole pI range from 4 to 11. About 15 well-defined IgG reactive proteins were found between 20 and 28 kDa.

Seven IgE-binding protein zones, likely to be isoforms of the same protein in each zone, were evidenced with the serum #60 (Figure 4, C). The most acidic IgE-binding proteins (zone 1, pI 4.5-5.0) showed a series of 3 isoforms with a Mr range of about 70-85 kDa. In a 2^nd^ zone (pI 5.2-6.5), an IgE-binding protein with 7 isoforms was detected at about 65 kDa and another with 2 isoforms at 45 kDa (zone 3, pI 5.9-6.0) which might correspond to Zea m 22. The IgE-binding proteins of zone 4 were strongly recognized and had 4 isoforms (pI 6.8-7.6, Mr 45 to 50 kDa). They could correspond to the polygalacturonase Zea m 13. Three single spots IgE binding proteins were also detected at Mr > 94 kDa (zone 5, pI 7.3) and in the basic region (pI 10.6), at 50 (zone 6) and 30 kDa (zone 7). These proteins might correspond respectively to Zea m 4 (reticulin oxidase) and Zea m 1. The seven boxes defining the IgE reactivity zones were copied on Figure 4, A, B. Obviously the same proteins were recognized by patient's IgG and IgE Ab although some differences in binding intensity were observed. For instance the proteins in zone 3 were poorly recognized by IgE and strongly by IgG whereas proteins in zone 5 are equally recognized.

## Discussion

In a general approach to evaluate grass pollen allergy in Madagascar, we clinically and immunochemically explored in a previous study, the IgE reactivity patterns of 65 PAP suffering from symptoms during grass pollination season against six different wild grass pollen [7]. Since wild and cultivated grasses belong to the same Poaceae family, we worked herein on pollen from the 2 most important cereals cultivated in Madagascar: maize and rice. The allergenic sensitization to maize or rice pollen has been reported [12, 30] but was shown to be lower as compared to wild grass pollen partly due, at least for maize pollen, to a larger size and heavier weight than wild grass pollen grains resulting in a poor air-borne dispersal [31]. In some agricultural regions of Europe [13], the prevalence of sensitization (evaluated by SPT) of PAP attending departments of allergy in hospitals between March and June is about 16% in region where maize is cultivated in medium to high density and was up to 88% for wild grasses. We showed here, by SPT, that the sensitization to maize or rice pollen in the highlands of Madagascar is very high, reaching 60% of patients selected upon clinical symptoms during grass pollinating season. The prevalence of sensitization to 6 different wild grasses tested individually by SPT were reported to be of the same order of magnitude, between 45 to 55% when considered taxon per taxon [7] but 71% for any wild grass pollen. Thus, the epidemiological data are somewhat different with what was found in Europe. Although SPT data might be in favor of cross-reactivity between maize and rice pollen, this trend was not confirmed by immunoblot results that showed more reactivities to maize pollen (85%) than to rice pollen (40%). Since allergen families are shared between maize and rice as well as between wild and cultivated grass pollen, cross-reactivities were expected [16, 17, 24, 32–34]. However, some specific sensitization can also occur in maize pollen allergy and rather associated to occupational allergy [14, 15]. An absence of cross-reactivity is thus in favor of a genuine specific sensitization as a consequence of a sustained and specific exposure. The IgE bindings observed by immunoblot reflect clinically relevant and irrelevant IgE reactivities whereas SPT evaluates IgE reactivities able to induce mediator release, and thus being closer to clinical relevance. Whether maize (pollen and/or corn kernel) could induce more clinically irrelevant IgE-binding protein as compared to rice (pollen and/or grain) has not yet been studied. Interestingly the high immunoblot reactivity against maize pollen was observed within IgE as well as IgG isotypes whereas in 12% of patients, anti-rice pollen Ab response seemed to be restricted to IgE isotype. This may reflect differential sensitization processes.

Cross-reactivities also exist between pollen and grain food that may lead to potential food allergy symptoms. Indeed, 47.7% of the PAP studied herein also display food-allergy symptoms (Table 2) and 43% express IgE against proteins from ground rice extract (unpublished data). Fonseca et al [35] showed different IgE binding proteins profiles in the maize seed extracts depending of the cultivars. In our study, on pollen extracts from 5 selected maize cultivars, only small differences in IgE binding protein profiles were visualized. Consequently, it appeared worthwhile to use the largely cultivated local *Meva* cultivar for 2D separations. Regarding maize pollen proteins and allergens, very few 2D separation studies were available till now [15, 32]. The IgE binding pattern of the studied serum showed a larger coverage of the maize pollen allergens than what was reported with sera from PAP exposed to maize pollen in greenhouses [32]. Four IgE binding proteins detected could correspond to already characterized allergens: i) the very abundant Zea m 1, known as major pollen allergen of maize [22] and reaching 4% of the extractable protein content [36], ii) the polygalacturonase Zea m 13, iii) Zea m 4, a reticulin oxidase and iv) Zea m 22, an enolase also present in maize seed [35]. Allergens belonging to these protein families have also been described in some other wild and cultivated grass pollen [24] (www.allergome.org). Beside these 4 known proteins, described as the most prominent maize pollen allergens [16, 24], our 2D immunoblots revealed 3 other IgE binding protein zones that remain to identify. No indication of group 5 allergens was evidenced in agreement with some report claiming its absence in maize [34]. Controversially, on its own, the study by van Ree et al [37] showed that group 5 allergen is present in *Zea mays* pollen. Furthermore, links between Lol p5, Ant o 5, Phl p 5, Sec c 5, Phr a 5, Fes r 5 and Dac g 5 were noted by Mohapatra et al [38], with dominating Pooideae species, compared to Arundineae (Phr a 5) and Panicoideae (Zea m 5). Group 2 allergens were also questioned in maize pollen [34] and, however, reported upon DNA sequencing [39].

Our study suggests that cereal pollen allergy - mainly for maize one - should be taken into account more carefully. Indeed, SPT and IgE immunoblots results revealed a high prevalence in Madagascar similar to what is observed for wild grass pollen. Several hypotheses may be raised to understand the results. Firstly an increase of exposure to maize pollen may be incriminated. In Madagascar, like in many other African countries, the part of the maize as cereal feeding source permanently increased. Between 1970 and 2000, the increase of cultivated surfaces was more than 100% for the maize vs. 45% for the local *Sorghum* [40]. Moreover, peri-urban and urban agricultures are determinant in food supply for many huge cities [41] and in Antananarivo, people are living very close to corn fields used for supplemental food supply. Population is, consequently, more exposed to maize pollen source. In such a way the risk of allergenic sensitization is higher even for non atopic individuals. Indeed, each maize plant produces copious pollen and even though the size of the grains (around 100 µm) could limit its dispersal, it can reach more than 1000 m downwind from the maize crops sources. In most of the cases maize pollen deposition is around 200-400 meters depending of micrometeorological conditions and height of the source [31]. Environmental context is thus of high importance for such grass pollen allergy.

Secondly, adjuvant environmental factors like pollution may be incriminated in the observed increased sensitization. Like many huge cities, Antananarivo is deeply polluted (http://siteresources.worldbank.org). Out of 215 towns around the world classified according to - among other parameters - air quality, Antananarivo was at the 213^th^ position in 2007 and 209th in 2012 (www.mercer.com). Air quality measurements from 1996 (INSTN, Madagascar) already attested that, in different parts of the city, the concentrations of lead particles and those issued of combustion exhaust gases (< 10µm) were over WHO recommended standards. The impact of pollution on cultivated plants (www.sei-international.org) and on the increase of allergic disorders is now well documented [42]. Gas and particle pollutants were reported to act on pollen grains, their sub-fragments and on proteins themselves [43, 44], altering both inflammatory and immune responses of individuals and exacerbating allergic response [45].

## Conclusion

Several allergenic effects of the pollen issued from the main 2 crops cultivated in the highlands of Madagascar are attested by our study. Some of the main already characterized allergens from both pollen extracts were shown to be recognized by IgE from PAP. Numerous co-reactivities against wild grass and cereal pollen were evidenced. However, specific reactivities were also highlighted, especially for maize pollen allergens. The high prevalence of maize pollen sensitization in this region is likely related to the specific environment characterized by i) a proximity of the population to the allergenic source and ii) a highly polluted urban atmosphere. Such study should help to a better understanding of maize pollen allergy at a broader scale, for different regions in Africa and worldwide, with close environmental and agricultural contexts. The presence of dense urban and peri-urban agriculture could be a high environmental risk factor for people sensitive to maize pollen.
